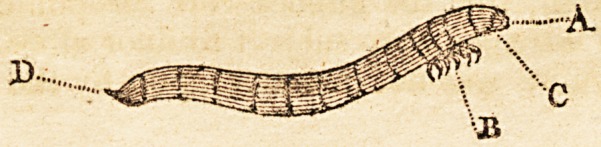# Mr. Custance's Case of Vertigo and Epilepsy

**Published:** 1804-05-01

**Authors:** Geo. Custance

**Affiliations:** Kidderminster


### *To the Editors of the Medical and Physical Journal.*

GENTLEMEN,

I Herein send you a drawing of a very curious insect, an account of which, I shall be glad if you can admit into the next number of your useful Miscellany. As it may possibly draw forth the observations and opinions of some of your Correspondents, I shall not commit myself by giving ing any opinion of my own; but only state such circumstances as I could collect from a narrow investigation of the subject.

On the 27th ult. Nurse L. of this town, took an emetic of her own accord, to relieve her of a giddiness. The emetic occasioned considerable efforts to vomit, without bringing any thing up but the warm water which she drank during its operation. At length, from straining, the left eye and nostril suddenly swelled, and about a tea-spoon full or two of blood flowed from the nostril, followed by the insect, which fell upon the hearth, besmeared with blood. It was alive, but soon died. Both Nurse L. herself and a neighbour who stood by, *believe* the insect came from the nostril; though they cannot undertake positively to say, whether it might not come from the stomach in vomiting. However they would not hesitate to make oath, that it came either from the mouth or the nose.

That your readers may form their own judgment of the matter, it is necessary to state, that Nurse L. has been subject for about seven years to sudden vertigos, with a considerable pain at times across the top of the nose, which were succeeded by a fit of epilepsy, one of which she had on the day she took the emetic. About six years ago, when attacked in this way with vertigo, she hung herself, and was taken down apparently dead. I attended her at that time, and restored her by the usual means. Within these few days, I have questioned her as to the cause of her commiting that rash act; and she declares that she can give no other reason, than that she *"did not know what she was about."* She was constantly picking the left nostril, but it does not appear that she had ever any *particular* disposition to sneeze. It must also be remarked, that she frequently complained of a pain in her stomach, and is a woman much given to intoxication, to which cause her giddiness and fits have been commonly attributed.

The person who took a drawing of the insect, viewed it in a vial of spirits of wine, in consequence of which the figure here sent is somewhat magnified. The insect itself is not quite an inch in length and rather less in circumference than the figure appears. The colour is a light straw, its length is divided by twelve rings of a darker shade, and at irregular distances.

I refer

I refer your readers to Vol. 6. of the Edinburgh Medical Essays, for an account of two centipeds discharged from the nose, one of which is said to have been in the *frontal sinus;* but we are not informed how that fact was ascertained. I am, &c.

*Kidderminster, March* 8, 1804. GEO. CUSTANCE.

P.S. This insect, when viewed through a microscope, is found to be very hairy about the head and tail, and its feet to have each a black *claw.*

Explanation of the Drawing.

A. The head. B. Six feet. C. Two short antæna. D. Uncated projection from the tail.

## Figures and Tables

**Figure f1:**